# Highlighting: A Mechanism Relevant for Word Learning

**DOI:** 10.3389/fpsyg.2012.00262

**Published:** 2012-08-14

**Authors:** Hanako Yoshida, Joseph Michael Burling

**Affiliations:** ^1^Cognitive Developmental Lab, Developmental Cognitive Neuroscience Initiative, Department of Psychology, University of HoustonHouston, TX, USA

**Keywords:** highlighting, cued attention, early word learning

## Abstract

What we attend to at any moment determines what we learn at that moment, and this also depends on our past learning. This focused conceptual paper concentrates on a single well-documented attention mechanism – highlighting. This phenomenon – well studied in non-linguistic but not in linguistic contexts – should be highly relevant to language learning because it is a process that (1) specifically protects past learning from being disrupted by new (and potentially spurious) associations in the learning environment, and (2) strongly constrains new learning to new information. Within the language learning context, highlighting may disambiguate ambiguous references and may be related to processes of lexical competition that are known to be critical to on-line sentence comprehension. The main sections of the paper will address (1) the highlighting phenomenon in the literature; (2) its relevancy to language learning; (3) the highlighting effect in children; (4) developmental studies concerning the effect in different contexts; and (5) a developmental mechanism for highlighting in language learning.

The order in which we acquire information matters to perception and learning. What is experienced first influences the perceptual (and conceptual) interpretation of what comes next (Duncan, 1980; Medin and Bettger, 1991; Shanks, 1992; Lamberts and Kent, 2007; Ramscar et al., 2010), and it does so in part by guiding attention (Kruschke, 1992, 2001; St Clair et al., 2009; see Ramscar et al., 2010, for a discussion and review, and also Yoshida and Smith, 2003). In this way, past learning constrains new learning. In this conceptual paper we consider a particular phenomenon known as the highlighting effect. This learning effect has been widely observed with different stimuli and procedures and in several domains (and has been developed within the framework of classical conditioning, associative learning, and problem solving), yet it has not been considered with respect to language learning and it is not well studied in children. However, the phenomenon is an instance of a powerful learning effect, such that temporally ordered cues that contain some predictive value influence what we attend to and what is learned. Therefore, the phenomenon may be a domain-general mechanism that plays a critical role in how previous language experiences help direct and facilitate new learning.

A key contribution to the study of the highlighting effect considers how this type of learning can explain seemingly domain-specific mechanisms. In addition, the phenomenon may be the foundation of an overarching theoretical framework for studying the developmental mechanisms that play a role in early cognitive achievement. To bridge the gap in the literature, we first introduce the highlighting effect by reviewing the typical task structure used in experiments with adults and then relate the effect to the process of word learning. Second, we discuss the potential importance and plausibility of cued attention processes in both linguistic and non-linguistic domains to demonstrate that developmental work may shed light on the way in which previous learning influences attention and learning for later information processing.

## Highlighting in the Literature

Highlighting is a mechanism that depends on both weighting cued attention and memory. The traditional highlighting task used with adults (e.g., Kruschke, 1996, 2005) works by presenting information about cues and outcomes in two phases. The structure of this paradigm relies on the notion that information is presented sequentially with some inherent overlap. Imagine that a learner initially learns that when “clouds” and “high humidity” appear on a computer screen, the learner will see “thunderstorms” as an outcome. The learner subsequently sees “clouds” and “low humidity” appear on the screen, and “nice sunny day” appears as an outcome. Notice that “high humidity” is a perfect predictor of “thunderstorms,” and “low humidity” is a perfect predictor of “nice sunny day,” whereas “clouds” is an imperfect predictor. Thus, there is symmetry between the two responses, each having a unique perfect predictor and sharing an imperfect predictor. Given the simplicity of this structure, it is reasonable to assume that a person receiving information structured this way will learn the symmetry. This assumption can be tested by observing the person’s decision about outcomes after seeing the single cue word “clouds.” If the person has learned that the cue is an equally imperfect predictor of the two outcomes, then the person should respond with the two outcomes with equal frequency. However, if learners are presented with some information before other information, their learning is not symmetrical. To use our weather example, if learners are first presented with “clouds and high humidity predict thunderstorms” and later presented with partially overlapping conjunctive cues and a new outcome, then the results across many highlighting experiments suggest that learners will take “clouds” alone as predicting the first outcome with which it was associated, in this case, “thunderstorms,” even though it is an equally imperfect predictor of the two outcomes.

**Figure 1:**
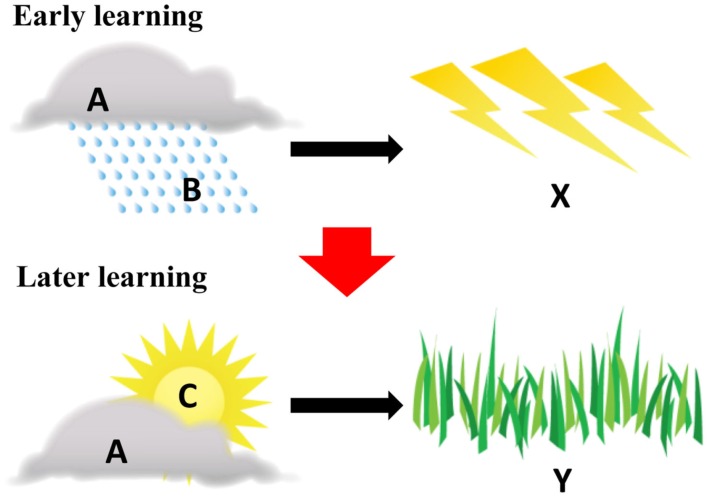
**Structure of a highlighting example**. A, B, and C (clouds, high humidity, and low humidity, respectively) are predictive cues. X (thunderstorm) and Y (nice sunny day) are outcomes. The broad red arrow signifies the order of learning (e.g., first Early learning, then Late learning).

The cue-outcome structure will be described in this paper with generic notation: A, B, and C for predictive cues, and X and Y for outcomes. Figure 1 displays this notation in accordance with the hypothetical weather example. In a typical highlighting task, interspersed training of A·B → X and A·C → Y continues until both are learned well, and adult learners often learn the cue outcomes nearly perfectly. Of interest are learners’ outcome choices in ambiguous situations, such as when they are shown clouds only, or rain and sun in conjunction. When probed with Cue A by itself, people are not impartial, instead strongly preferring Outcome X. On the other hand, when presented with the cue pair B and C, people strongly prefer Outcome Y, even though this combination was not presented during training. This bias in people’s preferences, going one way for A but the opposite way for the B·C combination, is the highlighting effect. Attentional explanations of the preferences suggest that the effect emerges during the later learning phase; that is, Cue C is attentionally highlighted during the learning of the case A·C → Y: the learner’s attention is shifted from A to C, because A has already been associated with X during earlier learning (Kruschke and Blair, 2000; Kruschke, 2003) and the competition from this association shifts attention to the novel cue. This attentional bias for Cue C generates a stronger association with the also novel Outcome Y. Notice that the highlighting effect in later learning guides learning about novel information in the moment and also helps learners retain earlier learned associations.

Consistent results have been documented in studies that tested different ratios of early and late learning, such as alterations of the fixed three-to-one base rates used in early studies (Lamberts and Kent, 2007), dual-task implementations (e.g., Medin and Bettger, 1991), and time restrictions placed on outcome choices (Shanks, 1992). Given the degree of stability in replications across different conditions, the validity of observed response biases is not in doubt; the existence of such asymmetry is widely accepted. Further, although the phenomenon is well-documented, it requires careful experimental controls to rule out the possibility that primacy and pure probability effects are in play, rather than strictly order of information effects. In adults, research on highlighting has typically focused on decision making in which outcomes are chosen based on asymmetrical learning of training items – asymmetrical in the sense that specific cues are given more weight over time, despite the symmetry provided by the training structure (Kruschke, 2003; see also, the inverse base-rate effect; Medin and Edelson, 1988). Moreover, the mechanism that produces the unequal weighting of cues during late learning has still not been determined, although a strong contender, and the one on which we focus here, is cued attention.

Cued attention that changes as a function of learning is fundamental to modern cognitive theories, such as the general context model of categorization (e.g., Nosofsky, 1986). In connectionist models, learned associations update connection weights, making some sources of information more important than others through cues competing with one another for relevance, but this produces learning patterns that often differ greatly from those that would arise by simply recording the correlations between cues and outcomes (e.g., Rescorla and Wagner, 1972; Rescorla, 1988; Kruschke, 1992, 2001). That is, within these models, cues interact and compete. The outcome of this competition can be understood in terms of changes in attention that merge broader sources of learning so that early and later learning together influence the attentional weights (Medin and Schaffer, 1978; Medin et al., 1982; Kruschke and Blair, 2000; Kruschke, 2003). A cued attention model that predicts learners’ attention to the most predictive cues would do a very good job of explaining a wide range of learning (see Yu and Smith, 2011), yet what more advanced learning models – and the highlighting phenomenon – suggest is that learning is not simply a matter of co-occurring cues and outcomes but is about a system of cues and outcomes, and that the order in which those cues are experienced is critical, along with sufficient memory capacity to retain such information. Earlier cues have a kind of precedence and are not easily overturned, because they shift our attention to what is not yet learned.

This shifting effect depends critically on competition, which is at the heart of modern theories of cued attention more generally (e.g., Desimone and Duncan, 1995; Duncan, 1996). For example, the biased competition theory of selective attention (see Desimone and Duncan, 1995; Duncan, 1996; Beck and Kastner, 2009) begins with the assumption that competition characterizes representational processes at the sensory as well as the cognitive level. Attentional selection occurs by biasing (e.g., priming) some representations over others. With respect to highlighting, reducing interference between current information and previously learned representations is thought to be achieved through biasing attention away from ambiguous cues and strengthening associations between more informative ones.

## Highlighting and Word Learning

Ramscar et al. (2010) provide a detailed account of word learning and label ordering in which they suggested that cue competition and order effects play a vital role in extracting meaning from labeled objects. As the highlighting paradigm’s primary focus is to consider the role that order has on learning across multiple experiences, Ramscar et al. concentrated on how cue competition aids in the learning of labels given an initial set of features (cues) from which to abstract information. This prediction, that a set of features can lead to a specific outcome (e.g., cues A·B, which can stand for any type of paired cues, such as color, shape, gestures, etc., leads one to predict Outcome X), is thought to involve discriminatory processes that span multiple learning instances. Encountering features first before hearing labels provides greater discrimination among cues and better prediction of the label as opposed to hearing a label and trying to predict its corresponding set of features. All relevant information regarding the features of a label is utilized in the moment to predict the meaning of that label. But if this procedure was reversed, that is, if successful understanding of the correspondence between cues and label was solely dependent upon the heard label (label X predicts cues A·B), it would be a much more difficult task, given that there would be so little source information to work with.

The nature of what actually constitutes a cue or outcome is not of primary concern in the learning simulations portrayed in Ramscar et al. (2010). Features of an object are either present or absent, but the option to consider the saliency of certain items may be implemented. The focus of their work is on the amount of information provided in the moment – and the degree of competition – that can help the learner successfully deduce and abstract some other level of understanding in the long run. The acquisition of labels is heavily influenced by the wealth of information abstracted from the environment.

The highlighting paradigm is also concerned with many of the same influences, such as cue competition and cue/outcome ordering, but it is even more focused on the mechanisms that are shared across multiple learning situations, which may or may not contain overlapping information between time points. Rather than referring to feature discrimination without much regard for temporally sensitive information, the highlighting phenomenon is based on a specific set of overlapping cues and outcomes that serve as a foundation for understanding the mechanistic components involved in asymmetrical learning. These cues are presented in a specific order with collapsed information during early learning, and new combinations with some overlap are presented at a later point in time. This temporal structure allows for a more detailed analysis and control over the influence of ordered learning effects, a factor not specifically designed for in the Ramscar model. In addition, the highlighting paradigm proposes that cued attention is the driving force behind the evaluation-updating cycle taking place when one is provided with a set of potentially informative cues that can be used to come to some conclusion about the world. Yet still, relatively little is known about the interactive processes involved between temporal factors and attentional development in children – especially how the order of perceived information contributes to constructing certain types of biases – and the underlying capacity for attentional flexibility at a given period in cognitive development.

Three ideas drive the present interest in highlighting as a mechanism in early lexical learning: the first is that words cue attention. That is, because words systematically co-occur with different information sources and perceptual events, words can serve as systematic cues for attention (e.g., Huetting and Altmann, 2007). As a general construct, attention and cued attention are widely considered to be important to early word learning (Plunkett, 1997; Hollich et al., 2000; Smith, 2000; Golinkoff and Hirsh-Pasek, 2006; Pruden et al., 2006; Yoshida and Hanania, 2007; Halberda, 2009); and infants show early sensitivity to contextual cues as guides to attention and learning (Tomasello, 1995; Saffran et al., 1996, 1999; Saffran, 2003; Ramscar et al., 2010; Nomikou and Rohlfing, 2011). More specific to the attentional role of speech, research on on-line speech processing by adults shows strong attentional effects of words cuing attention: words appear to automatically direct looking to the location of a mentioned object (Altmann, 2004; Knoeferle and Crocker, 2007).

**Figure 2:**
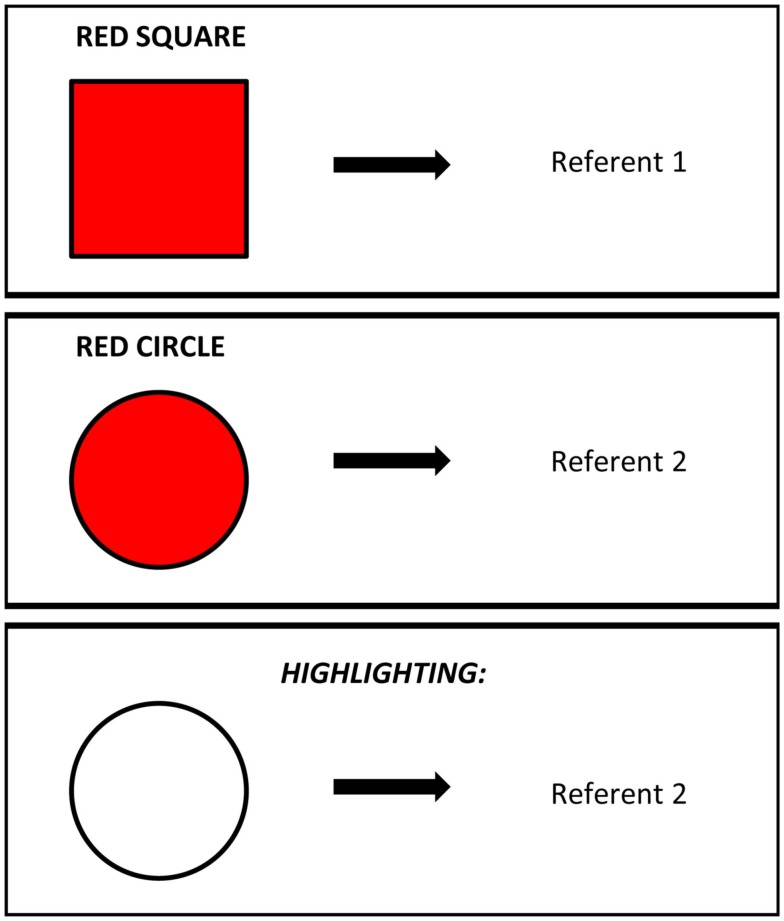
**Example of referential prediction via highlighting**.

The second key idea is that language is an ordered series of words. Because words are ordered in sentences, the co-occurrence of words is a possible conjunctive cue that can serve to organize attention in the moment to direct new learning. For example, highlighting provides a possible mechanism for referential disambiguation when there are multiple words and referents to be learned. Briefly, if a learner is first taught that RED SQUARE predicts Referent 1 (a single frame of reference between a set of features and a label) and is then taught in a later frame that RED CIRCLE predicts Referent 2 (see Figure 2), highlighting predicts that the learner will not learn about the relationship of RED and Referent 2 but rather will selectively attend only to the novel component CIRCLE and will learn that CIRCLE names Referent 2. That is, given the right history of frames and words, a novel word in a known frame should be associated with the novel candidate reference (in this case, shape). A large literature in early lexical learning for both nouns (e.g., Soja et al., 1991) and verbs (e.g., Gleitman, 1990; Fisher et al., 1994; Hirsh-Pasek et al., 1996) indicates the strong role of the frames in which novel words are presented in directing attention to the right meaning. There are many potential explanations of these results (e.g., syntactic bootstrapping, Gleitman, 1990). But, it may also be useful to consider this phenomenon in terms of the highlighting mechanism, as highlighting might provide a means to explain the strong role of high-frequency frames in directing attention to novel words and novel referents, even in very young (i.e., 14 months old) word learners (Fennell and Waxman, 2010).

The novel cue to novel outcome nature of the highlighting phenomenon coincides with considerable empirical evidence showing a strong tendency to map novel labels to unfamiliar items (e.g., Markman and Wachtel, 1988; Merriman and Bowman, 1989; Markman, 1991; Horst et al., 2011). For example, when 24-month-old children are shown a known thing, such as a bottle, and a novel, never-before-seen thing and asked “where is the *zop*,” they look to or retrieve the novel object. This widely replicated phenomenon is called “fast mapping” (Carey, 1978), as if children fast map the novel word to the novel object. As illustrated in Table 1, the structure of this so-called mutual exclusivity looks very much like that of highlighting: a child learns a cup by being presented with the referent, CUP, in a naming context. Let us call this learning instance (cup + naming context → CUP) the child’s previous learning. This is then followed by a subsequent naming context with a new cue (the novel word) and outcome (the novel object). In terms of highlighting, the child’s preference for mapping the novel name to the novel object may be the effect of the previously learned association (CUP + naming context → the word cup), as if the old cue (in a conjunctive cue setting) highlights the new one.

**Table 1:** **Parallel structure of highlighting and noun learning**.

Highlighting	Noun learning
Cue A + Cue B predicts outcome X	*Naming context* showing *CUP* predicts “cup”
Cue A + Cue C predicts outcome Y	Naming context showing *novel object* predicts *novel name*
Cue A is associated with outcome X more than with Outcome Y; Cue C is associated with outcome Y more than Cue A is	*Naming context* is associated with previously learned word “cup”; *novel object* is associated with the *novel name*

Yet the phenomenon itself may be a more in the moment attentional phenomenon. Horst and Samuelson (2008) have shown that children – given just one such fast mapping trial – do not remember the mapping when tested 5 min later. A recent study has shown that prior experience with objects enhances the remembering of newly learned labels for the objects (Kucker and Samuelson, 2011). From a highlighting perspective, overlapping frames in which the known and the subsequently learned novel word are embedded might also be critical. This has not been systematically examined, but it is an example of how a general mechanism such as highlighting might reveal the way in which structured language systematically cues attention. This process happens in the current moment and aids comprehension and learning over time.

The third reason why highlighting might be useful for understanding the prowess of early word learning is because it is a mechanism that – through competition – protects past learning by solidifying the representation between reliable predictors and their respective outcomes. The prior learning of A·B → X (in Figure 1) pushes attention away from A, from what is known, in the case of A·C → Y. Competition between known and unknown has been recognized by a number of theorists of early word learning as a critical component of lexical learning. The importance of competition is evident in Clark’s (1987, 1997) work on competition among developing meanings, in Markman and Wachtel’s (1988) research on mutual exclusivity (see also, Markman, 1991; Merriman, 1999), and in Au’s (1990) approach to lexical contrast in semantic contexts. Lexical competition is also important in MacWhinney’s (1989, 2004) competition framework and Siskind’s (1996) model of statistical word learning. More recently, a growing number of child-language researchers have linked these ideas about competition in lexical development to the competitive processes that characterize on-line sentence processing (Halberda, 2009; Horst et al., 2010; Smith et al., 2010). The idea that competition, and more explicitly, highlighting, may play a role in lexical learning is being studied in adults (e.g., McClelland and Elman, 1986; Marslen-Wilson, 1987, 1990; Tanenhaus and Spivey-Knowlton, 1996; Gaskell and Dumay, 2003). The consensus in that literature is that the listener attempts to build a mapping from a stream of words to the potential referents (and potential meanings) within a current context.

**Figure 3:**
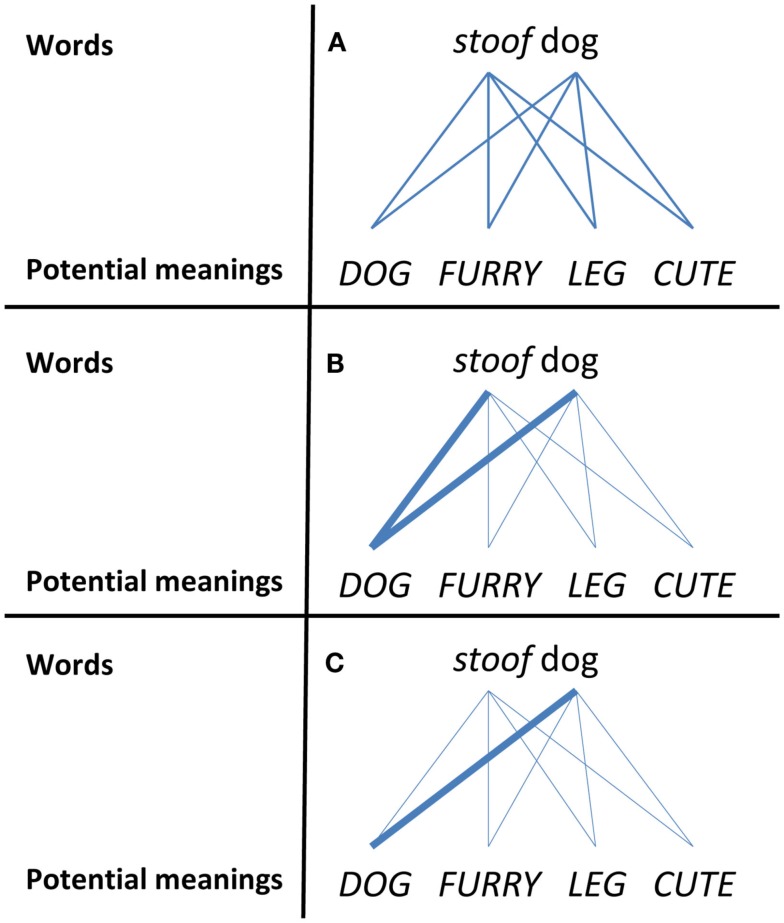
**Illustrations of how competition might develop (bold lines) and benefit early adjective learning**. **(A)** The mapping of words to potential meanings is equipotential for a novice learner. **(B)** The whole object constraint leads to a higher probability of mapping words to the object category over individual properties. **(C)** Partial exposure to the dog-DOG mapping leads to the inhibition of stoof-DOG and increased activation of dog-DOG.

Yoshida and Hanania (2011) used Figures 3A–C to illustrate how competition might benefit early adjective learning. Figure 3A shows the expectations for a novice learner who knows no words. For such a learner the mappings between all possible meanings might be considered possible. However, for young children, all meanings are not equipotential on both perceptual and conceptual grounds (Gentner, 1982; Markman, 1991), and given no other constraints, there is a higher probability of mapping words to the whole object category than to an individual part or property (Markman, 1991). Thus, as shown in Figure 3B, for a novice learner who knows a few words, labels might primarily compete for whole objects as their referents within a context. This is where competition might play a role. The usual assumption about this process is that inhibition is a function of magnitude of activation. Strongly activated cues simultaneously inhibit other cues. This reduces the probability that other possible candidates are also activated in conjunction. In this context, if the child knows – even partially – the dog–DOG mapping, this knowledge might work to inhibit a mapping from *stoof* to DOG, and the dog–DOG mapping may further develop through the process shown in Figure 3C and continue to generate consequential competition for future learning. These ideas have been tested in the context of adjective learning, suggesting that noun knowledge is an influential competitor in the adjective learning process. Sufficient activation of the noun helps to map novel adjectives to novel properties, an idea we will refer back to when discussing the potential relevancy of highlighting-like effects in the linguistics domain.

In sum, word learning – with its statistical regularities, with repeated frames and early and later learned words, with competition a major mechanism – is a likely domain in which an interdependent mechanism such as highlighting could play a role. But we must first ask whether children show highlighting effects in any domain, linguistic, or non-linguistic. Although highlighting itself is about cued attention – and thus seems to be a general mechanism that should be available to young children – the type and complexity of representations formed between cues and corresponding outcomes (e.g., simple visual cues vs. complex semantic representations) may reflect the child’s cognitive abilities and experiences at a given point in time, thus differing across developmental periods.

## Do Young Children Show Highlighting Effects?

The highlighting mechanism depends on attentional weighting and memory of previous associations, and the effect depends on order of learning and how strong initial predictive cues compete with later cues. How strongly does the first conjunctive cue need to be learned to compete effectively? How well do children form these kinds of cue-outcome associations? One finding from word learning tasks hints at the relevancy to language learning. Fernald et al. (2010) measured 2.5- and 3-year-old children’s gaze patterns when they heard familiar noun and adjective pairs (e.g., “blue car”). The key experimental variable was whether two potential visual targets differed in a property relevant to the adjective (“blue car vs. red car”) or the noun (“blue car vs. blue house”). The 3-year-olds were quickly able to use both the adjective and the noun to orient to the appropriate target, but the 2.5-year-olds’ attention with the adjective cue was much slower. Younger children know a considerable number of words and yet the cuing effects of words differ across different age groups. Studying the order effect provides insights into fundamental questions about how known words can help children learn new words: how much learning is needed for learned words to rapidly cue attention to a referent, and how strong does this old cue have to be to highlight novel information in subsequent learning? Are all cues equipotential, or does the learnability of a cue as a force on attention depend on past experience with that cue?

## Highlighting in Non-Language Tasks

Although there appears to be great potential in studying the highlighting effect in the language domain, there have been very few developmental studies of highlighting. Critically, the relevant question here seems not to be whether children show highlighting effects that share all the properties of highlighting in adults. Young learners have different perceptual experiences and have a different information processing capacity, and these may result in a different learning outcome. Rather, the relevant question is whether there are highlighting-like effects, and more specifically, whether order effects lead to asymmetrical biases that influence what is learned. The little evidence that does exist on highlighting in children suggests that the effect may not emerge in mature form until later development. For example, Winman et al. (2005) found that only one third of the tested school-age children (8–9 years old) showed the predicted effect.

**Figure 4:**
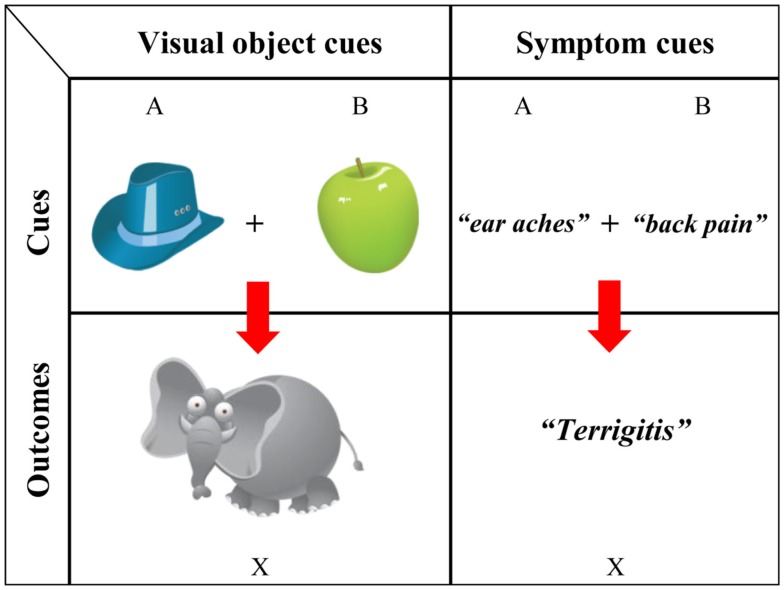
**Early learning from visually based cues (left) and inference-based knowledge (right)**.

**Figure 5:**
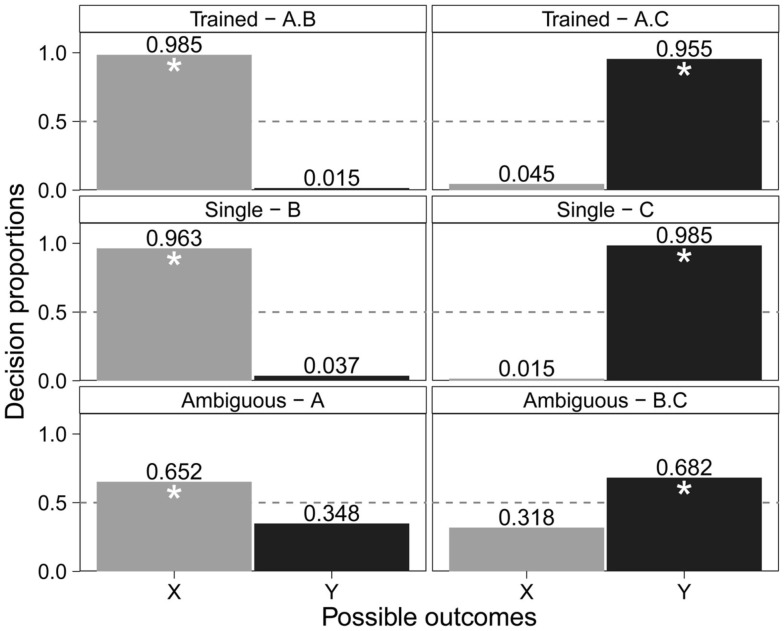
**Adult data from Burling and Yoshida (2011): decision proportions for possible outcomes (X, Y) given predictive cues (A, B, C) with the asterisks identifying reliably above chance (*p* < 0.05)**.

To better understand both the mechanism of highlighting and its development, we tested for early order effects by introducing a more child-oriented task in both child and adult populations (Burling and Yoshida, 2011). First, we compared different versions of base-rate and canonical highlighting tasks in adults to investigate possible differences between visually processed cues (Figure 4, left side), which we later used to test children, and inference-based knowledge (Figure 4, right side), as is often studied in adults. In our design, one of the tasks involved thematically linked category formation (e.g., Figure 4, left side: participants were instructed to learn which pairs of symptoms predicted a particular disease); testing probes were taken from Kruschke (2009). Another task involved visual exposure (e.g., Figure 4, right side: watching illustrated images of a hat and an apple move toward an elephant). The two cues presented at the top center of the screen always moved simultaneously and diagonally toward the outcome presented either on the bottom left or bottom right side of the screen. Different outcomes (animals) were presented in the early and late training phases and were associated with two cues (objects). One of the cues was present in both early and late training phases, while the other cue was unique to the specific phase. Cues presented during training, novel pairs of cues, and single cues were probed during testing. Participants were asked to choose which outcome corresponded to the given probes. The results are shown in Figure 5. Using the newly developed visual cues, we were able to replicate strong early and late learning in adults. Trained with cue pairs (Cues A·B and Cues A·C) and single cues (Cue B alone and Cue C alone), adult participants learned the cue outcomes nearly perfectly. The interesting finding was learners’ outcome choices in ambiguous situations (A alone or B·C). When probed with Cue A by itself, adult participants reliably selected Outcome X, but when presented with the cue pair B and C, they reliably selected Outcome Y. The data also suggest a relationship between how participants respond in the visual and typical highlighting tasks (correlation between the corresponding trials across the tasks was reliable, all above τ = 0.31, *p* < 0.01, and expected accuracy in both tasks was high). This relationship between tasks confirmed the new visual paradigm as suitable for measuring highlighting and established a procedure for use with young children. The finding of the effect in visual learning also fits the attentional interpretation of the phenomenon (Kruschke, 2003).

**Figure 6:**
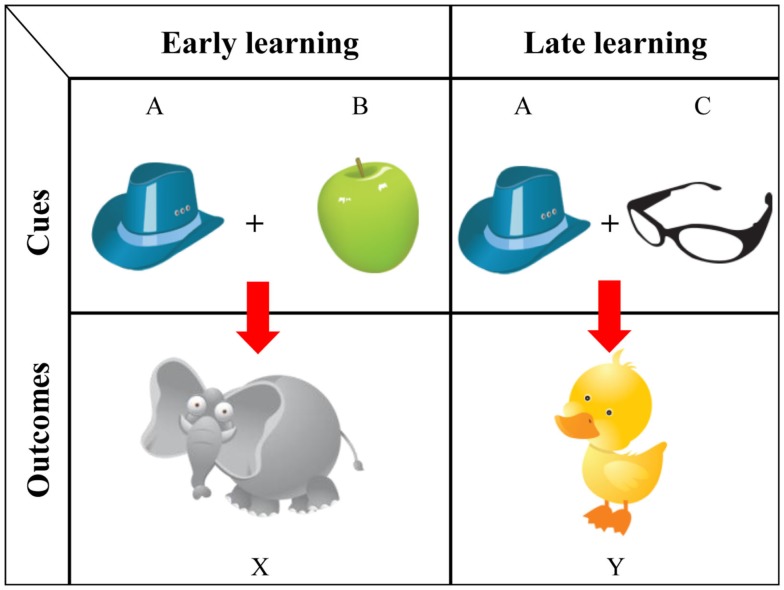
**Complete highlighting structure for visual stimuli used with children**.

**Figure 7:**
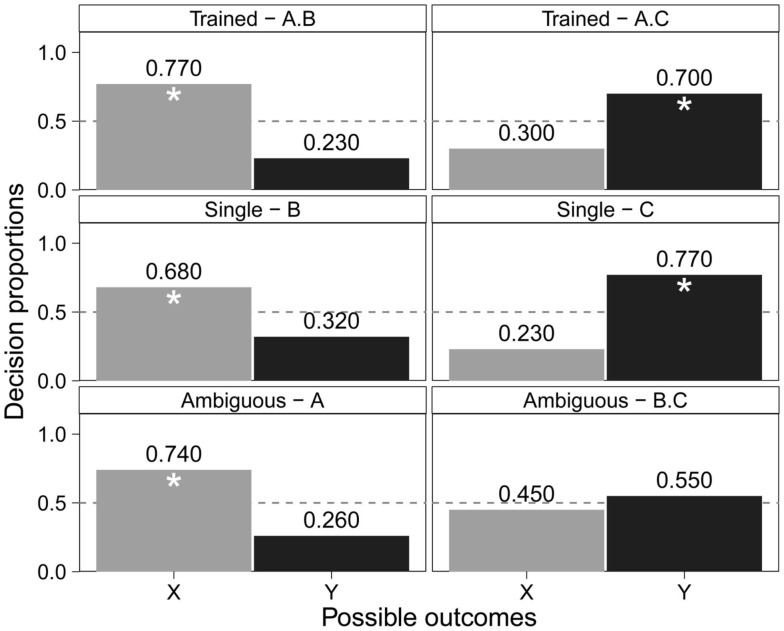
**Older children’s data from Burling and Yoshida (2011): decision proportions for possible outcomes (X, Y) given predictive cues (A, B, C) with the asterisks identifying reliably above chance (*p* < 0.05)**.

Next, we tested young children (3- to 4-year-olds) with the same visual highlighting task used with adults (see Figure 6 for the complete task structure). Previous attempts have been made to test children using base-rate information with the disease diagnosis structure designed for adults, but they failed to find evidence of asymmetrical learning in 8- to 9-year-olds (Winman et al., 2005). It may be that the results obtained from this study were due in part to the difficulty of the task itself for that particular age group. The pairing of visual cues and objects stays true to the highlighting paradigm while making the task more accessible to younger children. The results suggest that order effects do matter when certain sets of cues are presented before others; early and late learning created response biases in children during testing. As is evident in Figure 7, the older children (5-year-olds) learned both early (X) and late (Y) cue outcomes, and they also successfully selected expected single cue outcomes. These older children’s response frequencies were found to be similar to those of adults. However, the highlighting effect was only found in one of the ambiguous cues (A alone).

**Figure 8:**
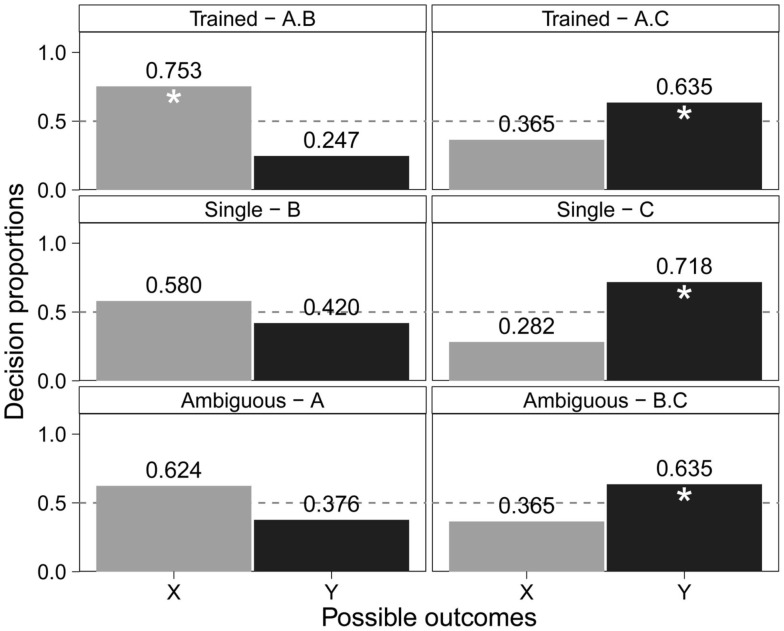
**Younger children’s data from Burling and Yoshida (2011): decision proportions for possible outcomes (X, Y) given predictive cues (A, B, C) with the asterisks identifying reliably above chance (*p* < 0.05)**.

In contrast, younger children (3- and 4-year-olds) showed relatively less learning from later training overall (younger children required more training trials than older children) and demonstrated the asymmetries characteristic of highlighting in primarily the ambiguous outcome of B·C. However, note that although they learned the highlighted cue, these young children failed to learn the single cue outcomes (B → X, and A → X). They were not able to choose the early outcome (X) significantly often when given Cue B or Cue A (see Figure 8). It is often possible to demonstrate the effect based on the asymmetrical responses for *both* ambiguous testing items. However, the response biases for these probes may involve incremental processes based on an individual’s current cognitive ability. The developmental differences observed may suggest that the highlighting effect is not all-or-nothing but involves gradual processing of information.

One interesting aspect relevant to early word learning concerns the revising process, or the ability to rapidly retrieve previous knowledge for updating based on more current information. For children to choose the early outcome (X) given the uniquely predictive early Cue B after the highlighting effect has emerged, they somehow need to revisit the early-learned A·B → X (i.e., A and B are equally predictive of X) and isolate B as a single predictive cue, because children might perceive A·B → X as meaning A·B is a single cue. The results with younger (and to some degree with older) children suggest that they may not revisit past learning in this way. For younger learners there is competition, and this competition might actually help them isolate the components, because they seem to learn about C in isolation. This could be due to the novelty effect, such that the novelty of C inhibits learning that A *also* predicts Y, so when asked about A alone, they overlook the fact that A has some role in predicting Y. If the novelty of C creates a stronger association with Outcome Y, the increase in saliency between the two cues as a result of this novelty might be more of an influential force for younger children than for adults. Previous research has shown that younger children are more sensitive to the saliency of cues (e.g., toys with salient features) in learning objects and over time become more sensitive to other sources of information, such as social cues (e.g., eye gaze; Golinkoff and Hirsh-Pasek, 2006). Thus, the strong association between C and Y may only weakly affect original learning (A·B → X) when given A·C conjunctively, but this C → Y highlighting effect is efficient for forming a prediction that B·C → Y, which suggests competition. Accordingly (without the novelty effect witnessed in late learning), younger children may not know what to do with B alone, since it was always seen in conjunction with A. The successful parsing of early cues as separate items involves not only the revisiting of these cues, which is also subject to the young learner’s memory, but also overcoming the strong novelty influence seen in later learning.

In contrast, older children may be better at revising early learned cues and learning in later training (thus generating a new form, such as B → X), but this learning seems to hinder their attentional biases toward Y given B·C, because of the competitive force of B → X. This suggests that the highlighting effect involves well-adjusted retaining and revising processes across time; the effect of early learning can continue throughout the learning period instead of just making the highlighting effect happen. In other words, older children are not as influenced by the saliency of C → Y and can thus segregate cues accordingly via an enhanced revising capability. The result is that older children have a stronger tendency than adults to give individual, perfectly predicting cues (such as B and C) equal treatment, an indication that for older children, this revising process is more important than the influence of sheer novelty. That different strategies are implemented at different ages suggests that children readily utilize the cognitive capabilities available to them given their stage of development, with a transition to more saliency-based learning, to being able to revise past learning in order to make assumptions about the nature of objects. Clearly, investigation of the learning effect in individuals with various cognitive capacities is important for us to understand the nature of asymmetrical learning. The associations and attentional biases observed between two static, visual images displayed on a computer screen (visual–visual biases, more generally) cannot currently be extrapolated beyond image-based associations but serve as a foundation for further study in the domain of language. Choosing an object-oriented design to illustrate the role of ordered learning is a necessary condition for establishing the effect in younger children. The biases may be more pronounced in the language domain since these cue-outcome associations are pervasive in language. And thus, highlighting-like order effects may be more robust in the context of words and referents than in arbitrary visual–visual associations.

## Highlighting in a Linguistic Context

To formally examine the potential role of the highlighting effect in children’s word learning, we sought a context in which we could be confident of the robustness of the early cue-outcome (words-referents) learning and in which the conjunctive cues (co-occurring words) for the early and later learning were also clearly present in the input given to the children. On these grounds, our first linguistic highlighting study examined the role of early-learned nouns in subsequent adjective learning. Learning of adjectives has been considered a difficult task for young children, and children learn many nouns before they learn many adjectives (see Gasser and Smith, 1998, for a review; see also Nelson, 1973; Gentner, 1978; Dromi, 1987; Jackson-Maldonado et al., 1993; Mintz and Gleitman, 2002), suggestive of the role of nouns in learning adjectives (e.g., Waxman and Klibanoff, 2000; Mintz and Gleitman, 2002).

Further, studies of children’s novel adjective learning have consistently shown that children 2–3 years old often fail in these tasks by attaching the novel adjective to the shape of the labeled thing or to the noun category rather than to a property of the object (Au and Markman, 1987; Heibeck and Markman, 1987; Au and Laframboise, 1990; see also Sandhofer and Smith, 2007). However, if children already know the name for a basic-level category, they are much more likely to link the new adjective form to a property of the labeled thing than to a noun-like category organized around shape or overall similarity (Markman and Hutchinson, 1984; Waxman and Gelman, 1986; Markman and Wachtel, 1988; Baldwin and Markman, 1989; Waxman, 1990, 2010; Waxman and Kosowski, 1990; Hall et al., 1993; Klibanoff and Waxman, 2000). These tendencies and the potential role of early-learned cues in new learning documented in the domain of novel/adjective learning are consistent with the lexical competition in which old words (cues) compete with new ones and thus push novel cues to being linked to novel aspects of the predicted outcome. If this is the case, then the explicit mention of the noun should increase competitive effects and promote associating the novel adjective to a property.

Previous research by Mintz and Gleitman (2002) showed just this effect: the presence of a noun (as in “the *stoof* boat”) helps with the learning of novel adjectives, in comparison to conditions in which a noun is not explicitly labeled (“a *stoof* one”). Most recently, Yoshida and Hanania (2011) conjectured that this role of the noun could be understood as attentional highlighting. Table 2 shows the role of the noun in adjective learning in terms of highlighting. As described in the table, a 2-year-old child usually learns elephant by seeing the combination of elephant-shaped things and the typical elephant texture (e.g., ROUGH). Let us assume that this is the child’s previous learning about an elephant before being brought to a laboratory and presented with an elephant-shaped thing with a novel texture and a novel name. In terms of the explanation used for highlighting, the child’s preference for mapping the novel name to the novel texture may be the effect of the previously learned association (e.g., elephant shape to the word elephant). As a first step in examining this idea – that the role of the noun might be understood as the old cue in a conjunctive cue that highlights the new one – Yoshida and Hanania used ambiguous syntactic information but provided explicit naming (“an elephant *vap*”) and compared the children’s performances to the performances in a condition in which no previously learned noun was explicitly given (“a *vap* one”).

**Table 2:** **Parallel structure of highlighting and adjective learning**.

Highlighting	Adjective learning
Cue A + Cue B predicts outcome X	Elephant shape + standard texture predicts “elephant”
Cue A + Cue C predicts outcome Y	Elephant shape + novel *stoof* texture predicts “*stoof*”
Cue A is associated with outcome X more than with Outcome Y; Cue C is associated with outcome Y more than Cue A is	Elephant shape is associated with word “elephant”; *stoof* texture is associated with word “*stoof*”

Yoshida and Hanania (2011) contrasted this hypothesis with an alternative interpretation of the role of the noun (Mintz and Gleitman, 2002). According to this alternative, the noun is important not because it is a cue to attending to shape but because it provides syntactic information. If this is right, “*vap* elephant” should be effective (and better than “*vap* one”) in directing attention to the novel property; but “elephant *vap*” should not be a clear case for providing the effective information. According to the highlighting hypothesis, however, “elephant *vap*” should work just fine, and any additional previously learned association should strengthen the attentional bias (guiding attention) because several learned cues (“elephant red *vap*”) are better than fewer cues at directing attention to a novel texture.

The key experiments examined this prediction with 2-year-olds by using an adjective learning paradigm used by Mintz and Gleitman (2002). Children were first presented with exemplars – three red objects that shared the same texture (rough surface) – one at a time. For example, an experimenter presented a red elephant made of a rough (and highly unusual) texture, pointed to the elephant, and then said (in a list format with pauses) “elephant-, -red-, -*vap*.” The same presentation was made for two other items (e.g., red rough fish and red rough car). Following the presentation, children were asked to choose the “*vap*” from two test objects, one matching the type in terms of shape and color (e.g., another red fish with incorrect texture) and one with the correct color and texture but different shape (e.g., a red rabbit with rough texture). In half of the trials two known labels and one novel label were presented as described above (e.g., noun, color word, and novel name as in “elephant-, -red-, -*vap*”) and in the other half of the trials, one known label and one novel label were presented (e.g., elephant-, -*vap*). This was to address the prediction about the number of labeled contenders influencing adjective learning (thereby directly testing the magnitude of competition). If previous learning is what generates competition for later learning (as in the highlighting effect), then increased activation of previously learned representations (e.g., both the object name and color name) helps guide attention to the newly introduced property (e.g., texture). The key findings suggest that children mapped the novel word to the texture reliably more often in the trials when there are more competitors than in the trials with fewer competitors, and children only performed significantly above chance in the trials with two known labels. This suggests that although young children often incorrectly associate adjectives to whole objects rather than to the properties of objects, explicitly mentioning familiar words which are already strongly linked to their referents decrease the likelihood of novel words being falsely mapped to these known referents (with the help of increased activation of previously learned representations). The study illustrates how fundamental processes of lexical competition in on-line word comprehension may give young learners a way to leverage known words in learning new words.

**Figure 9:**
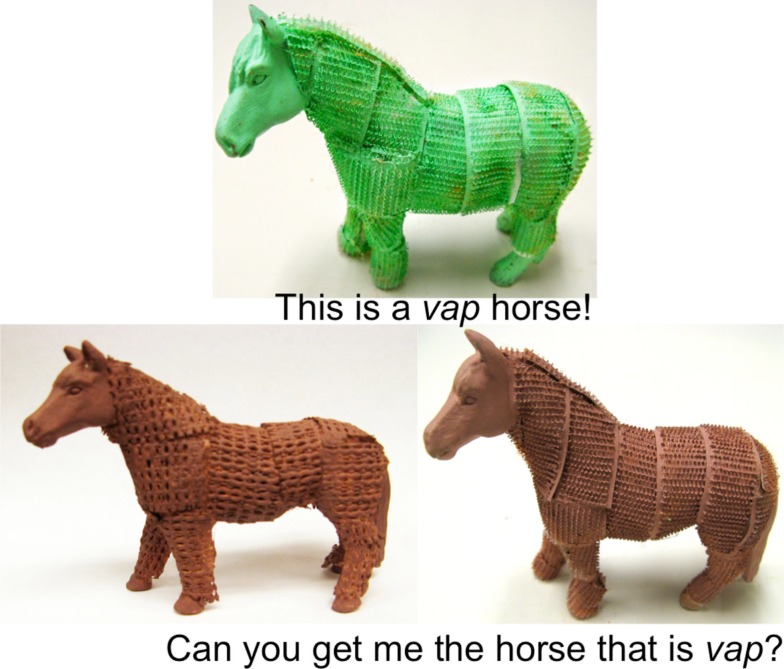
**The novel adjective learning task used in the study by Yoshida et al. (2011)**.

Yoshida and Hanania (2011) presented evidence suggesting a process similarity between highlighting and adjective learning, yet this framework depends on the assumption that flexible attentional shifting underlies the non-linguistic visual task and that the competition resolution in the word-mapping process is similar in nature. This leads us to ask if this linkage is really related to attention. There have been a number of studies suggesting that one key element of the word learning process is relevant to the control of attention – flexibly shifting to update with the current information on relevance – but there has not been any direct evidence supporting the linkage. To address this, we have recently documented the relation between children’s adjective learning performances and non-linguistic attention control performances (Yoshida et al., 2011). This study tested 3-year-old children with both a novel adjective learning task and a non-linguistic attentional network test (ANT; developed by Rueda et al., 2004) that measured attention control and analyzed the results for the relationship. The novel adjective task was very similar to those used in Mintz and Gleitman (2002), except there was only a single presentation of target property. Thus, it was considered a more difficult task (see Figure 9).

In the adjective learning task, the children were shown an exemplar object that was a member of a known category (e.g., horse) of a specific color (e.g., green) that also had the to-be-tested unfamiliar property (e.g., rough) and was told, “This is a *vap* horse.” The children were then shown two new horses. Both horses were a new color (e.g., both were pink) and one horse matched the target texture but the other did not. The two choice horses were pointed to and the children were then asked, “Can you get me the horse that is *vap*?” Their proportion of property-matching choices was analyzed.

**Figure 10:**
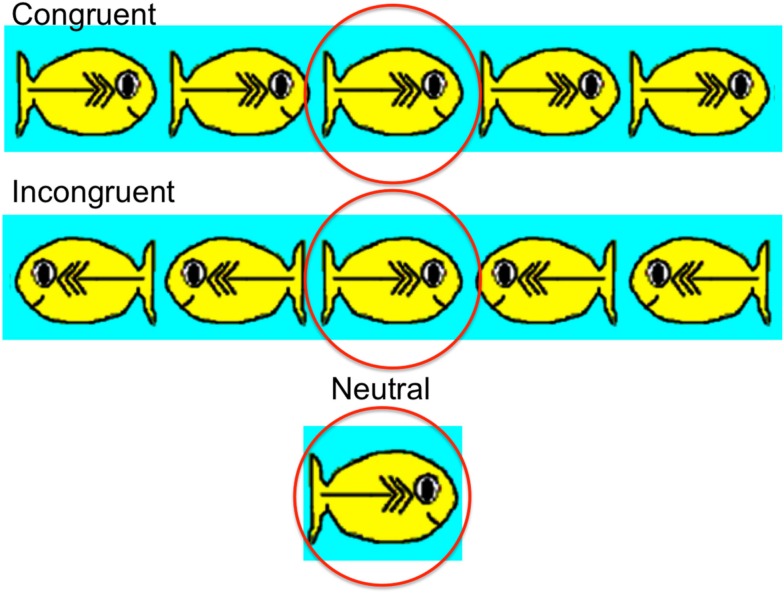
**The attentional network test (ANT) presentation in Yoshida et al. (2011)**. The target “hungry” fish are circled in red.

The ANT is a visual test (illustrated in Figure 10). In this study participants were presented with 36 test trials in which they were asked to feed a hungry fish (circled in red in Figure 10) by touching its mouth with their index finger as quickly as possible (the response was collected via a touch screen and analyzed for accuracy and speed later). A fixation point was presented throughout the task in the center of the screen. The target (i.e., the hungry fish) was either a single fish (neutral condition) or the middle fish in a row of five fish. The row of five fish could face left or right, and the stimulus could be in a congruent or incongruent direction (target fish facing the same or opposite direction as the other four fish, respectively). The results suggest that there were reliable overall correlations between performance in the novel adjective task and performance in the ANT, both in accuracy, *r* = 0.48, *p* < 0.05, and in response time, *r* = −0.50, *p* < 0.05. The findings provide the first direct evidence of a relation between performances in an artificial word learning task and an attentional control task, further supporting highlighting as an attentional phenomenon and its relevance to word learning.

## Building the Power of Language from Domain-General Mechanisms

Highlighting is a domain-general phenomenon, one that might emerge out of the competition between new associations that involve components of older, already learned associations. Despite being domain-general, it may build highly specific attention mechanisms that work in domain-specific ways. For example, a baby is first exposed to many unstructured individual associations (e.g., the word “dog” co-occurs with a particular kind of dog; the word “cat” co-occurs with a particular kind of cat in a picture book, and “chair” may co-occur with a specific toy chair with which the baby often plays). As the baby gathers many of these associations and repetitions, a few of the associations will emerge as being stronger – the words that refer to multiple but similar instances, for example. In addition, some level of activation among them may be generated (e.g., the word dog may activate commonalities among all other previous instances). These activations become organized over time to generate more targeted, systematic competition (e.g., given a word “dog,” different dog shapes may now compete in the mapping process). This ongoing competition supports constant evaluation, during which assignment of attentional weights is continuously revised and supported. This results in effective attentional biases being continuously updated and supported as the child encounters new task contexts that provide different types of cues (e.g., perceptual cues, word cues, etc.), and thus the relevancy of certain cues changes. The entire process can be considered in terms of a feedback loop. Information presented moment-to-moment continuously requests attention. As a result, this supports the development of both attentional flexibility and the competition process, which in turn influences where to attend. From this perspective, highlighting-like phenomena might be most critical for the evaluation process, which supports assignment of attentional weights based on temporal ordering of information. The specific function of such attentional flexibility appears similar to that of the weighting mechanism of the emergentist coalition model (Golinkoff and Hirsh-Pasek, 2006) that is broadly concerned with the variety of cues children use and the weights given to these cues, which shift across development. In this framework, the highlighting effect can be viewed as one of these domain cues, particularly temporal cues that influence learning differently at sequential stages of development. For example, a child’s developing memory capacity may partially account for observed learning differences and result in changes of memory guided attention toward specific cues.

The mature highlighting effect seems to be part of an evaluation process specifically geared toward generating attentional biases for new instances (and at the same time protecting old instances) given previously learned associations. In the case of word learning, a child might see a green apple and hear “apple”; both the apple’s shape and the green color are prominent, equally relevant features for the spoken word “apple” at this moment. Later, the child sees a red apple and then hears someone saying “red” in this context. Given the critical function of highlighting, in this later exposure the association between the color red and the word red is stronger than the association between the shape of an apple and the word red. This is due to the temporal nature of the two distinct learning experiences, in which attentional biases were already formed based on the retaining of the association between the apple shape and the word apple. However, the idea of such backward revision within the developmental literature has mostly been considered in the domains of causal relations among events and rule-based reasoning (e.g., Sobel et al., 2004; Winman et al., 2005; Sobel and Kirkham, 2006). If the aim is to evaluate the highlighting paradigm as a domain-general learning mechanism, we propose that cross-domain learning studies are needed in contrast to previous (and less general) attempts at backward revision theories among children.

Exposure to numerous associations sets the stage for competition processes, but the learning of associations becomes more powerful through the process of revising after some degree of learning has already occurred, with context-specific attentional weights being gained in the process (e.g., the role of learned words in learning new names). This illustrates that the amount of weight given to certain properties is the direct result of temporal order effects. It would be insufficient to consider all cues and associations equally via frequencies or probabilities exclusively. Only items relevant to the moment are considered, which facilitates future learning, and overlapping cues influence attentional weights, trimming certain associations while simultaneously strengthening others.

Certainly, learning strategies can differ at different ages. The current proposal does not assume that the mechanism generates the same types of biases for all age groups and across all task contexts. Highlighting, in particular, has been studied only with school-age children given a very specific set of task contexts, which are often far simpler than real-world language learning situations. The unique contribution of the present idea that the temporal sequence in which cues appear significantly affects learning, and the new findings suggesting that attention to these cues may change across developmental stages, is to offer a novel approach to describing how the process of cued attention can promote cognitive development. There is great potential to generalize this approach to broader contextual features such as the role of social interactions and phonological and linguistic elements.

Information is often given in sequences and thus it is critical to consider the order in which cues appear. Even simple associative pairings have an order, and the order effect has been documented by demonstrating that the characteristics of the first item (imagery value, concreteness, association value, etc.) are much more important than the characteristics of the second item in determining the level of performance in paired-associate learning (Paivio, 1971). The sequence of information acquisition is also important in more complex academic learning, such as mathematics. The National Council of Teachers of Mathematics (2000) has focused on *what* students should learn and *how* this learning should occur, and thus order – *when to learn what* – becomes a key addition to learning strategies. The implications of the current work are broad. Considering temporal factors in attention and learning has great potential for increasing our understanding of learning in various domains and at multiple levels.

## Summary and Future Directions

In the present focused conceptual paper, we concentrated on a single attention mechanism – highlighting – with the aim of presenting its relevancy to early word learning. Overwhelmingly, research in language learning has preoccupied itself with what children know and how this knowledge promotes developmental changes. The assumption has been – and largely remains – that children can only learn about what is directly in front of them (space and time are viewed as more static than they might be and thus interactions within the domain have been undervalued). As we have shown, both formally and empirically, this assumption is inconsistent with much of what we understand about the contribution of dynamic temporal factors. In the language learning context, we proposed that highlighting might serve to disambiguate ambiguous references, working hand-in-hand with competition processes.

Programmatic developmental work on these learning effects is clearly important, and the recognition of such effects in other domains will advance our understanding of the nature of human learning (via their relation to the development of working memory and attention) and the fundamental mechanism involved in children’s cognitive development. Last, research on general learning effects outside of language learning is also essential to understand how or if the nature of language learning differs from learning in other domains.

## Conflict of Interest Statement

The authors declare that the research was conducted in the absence of any commercial or financial relationships that could be construed as a potential conflict of interest.
